# P2 X 7 receptor is a critical regulator of extracellular ATP-induced profibrotic genes expression in rat kidney: implication of transforming growth factor-β/Smad signaling pathway

**DOI:** 10.1007/s11302-023-09977-4

**Published:** 2023-11-07

**Authors:** Fatma Mounieb, Somaia A Abdel-Sattar, Amany Balah, El-Sayed Akool

**Affiliations:** 1 Pharmacology and Toxicology Department, Faculty of Pharmacy (Girls), Al-Azhar University, 11751 El Nasr St, Nasr City, Cairo Egypt; 2 Pharmacology and Toxicology Department, Faculty of Pharmacy (boys), Al-Azhar University, Cairo, Egypt

**Keywords:** ATP, P2 X 7 receptor, Renal fibrosis, TGF-β/Smad signaling

## Abstract

This study was designed to investigate the potential of extracellular adenosine 5′-triphosphate (ATP) via the P2 X 7 receptor to activate the renal fibrotic processes in rats. The present study demonstrates that administration of ATP rapidly activated transforming growth factor-β (TGF-β) to induce phosphorylation of Smad-2/3. Renal connective tissue growth factor (CTGF) and tissue inhibitor of metalloproteinase-1 (TIMP-1) mRNA and protein expressions were also increased following ATP administration. A decrease in TGF-β amount in serum as well as renal Smad-2/3 phosphorylation was noticed in animals pre-treated with the specific antagonist of P2 X 7 receptor, A 438,079. In addition, a significant reduction in mRNA and protein expression of CTGF and TIMP-1were also observed in the kidneys of those animals. Collectively, the current findings demonstrate that ATP has the ability to augment TGF-β-mediated Smad-2/3 phosphorylation and enhance the expression of the pro-fibrotic genes, CTGF and TIMP-1, an effect that is largely mediated via P2 X 7 receptor.

**Supplementary Information:**

The online version contains supplementary material available at 10.1007/s11302-023-09977-4.

## Introduction

Adenosine 5′-triphosphate (ATP) is the main source of energy involved in all biosynthetic processes in cells. Once released outside the cell, the extracellular ATP as well as its metabolites participated in regulating various biological activities including muscle contraction, cardiac functions, neurotransmission, and hepatic glycogen metabolism [1].

The biological activities of extracellular ATP are mostly mediated via the P2X family purinergic receptors, which comprise P2 X 1-7 subtypes [2]. The purinergic P2 X 7 receptor (P2 X 7R) is an ATP-gated non-selective cation channel. Binding of extracellular ATP to P2 X 7R rapidly triggers the trans-membrane flux of Na, K, and Ca ions, tailed by the delayed induction of a non-selective pore that aids the permeation of molecules with large masses of up to 900 Da [3]. Among P2X receptors, the P2 X 7R seems to be the most involved in the inflammatory response. Indeed this receptor plays an undisputed fundamental role in inflammatory and infectious disorders [4]. It has been reported that P2 X 7 receptor is expressed in glomerular mesangial cells and podocytes [5, 6] and animal studies have provided evidences that link P2 X 7R expression and/or activation to renal diseases [7–9].

Transforming growth factor-β (TGF-β) has been extensively regarded as a crucial mediator of tissue fibrosis. It is highly expressed in a widespread range of kidney diseases accompanied in which the hallmark histological features is fibrosis [10, 11]. TGF-β stimulates the epithelial cell-to-mesenchymal transition (EMT) as well as extracellular matrix (ECM) proteins deposition via activating its downstream signaling pathways [12]. It is well recognized that TGF-β mediates fibrosis by inducing the phosphorylation of Smads and subsequent expression of pro-fibrotic genes such as connective tissue growth factor (CTGF) and tissue inhibitor of metalloproteinase-1 (TIMP-1) [13–15]. Also, the regulatory role of purinergic receptors in the fibrotic processes has been well documented [16, 17]. In this context, many studies have explored the key pathophysiological utilities of P2 X 7R in fibrotic diseases of lung, liver, pancreas and kidney [18–20].

The current work was designed to investigate the potential roles and molecular mechanisms by which ATP/P2 X 7R signaling could induce renal fibrosis in rats. The TGF-β1-driven canonical Smad-2/3 signaling cascade along with the expression of some pro-fibrotic genes have been examined in the presence of ATP and/or the P2 X 7R antagonist, A 438,079.

## Materials and methods

### Animals

Male adult Sprague Dawley rats (180-200 g) were acquired from Nile Co., Cairo, Egypt. Animals were caged in the animal facility of the Faculty of Pharmacy (Girls), Al-Azhar University (12 h dark/light cycle with precise control of humidity and temperature). Water and a standard diet were delivered *ad libitum*. They were harbored for seven days before the start of treatments for acclimatization. The experimental protocol was permitted by the Ethics Committee of Faculty of Pharmacy, (Girls), Al-Azhar University (Approval number: 177) in accordance with the standards of Principles of Laboratory Animal Care (NIH Publications No. 85 − 23, revised 2011).

### Materials

The P2 X 7 receptor antagonist, A 438,079 was from Sigma-aldrich Co. (St. Louis, MT, USA). ATP was from TSI USA, Inc. (Missoula, MT). TGF-β1 Quantikine ELISA kit (#DB100C) was from R&D Systems, Inc., a Bio-Techne brand (Minneapolis, MN, USA). Phosphorylated Smad-2/3 (p-Smad-2/3, #PA5-99378), total Smad-2/3 (t-Smad-2/3, #PA5-36125), CTGF (#PA5-32193), TIMP-1 (#PA5-99559) and β-actin (#MA1-140) antibodies were from Thermo Fisher Scientific Inc. (Waltham, MA, USA). Horseradish peroxidase (HRP)-conjugated anti-goat IgG antibody was from Sigma-aldrich Co. (St. Louis, MT, USA). Radioimmunoprecipitation (RIPA) lysis buffer was from Santa Cruz Biotechnology (Santa Cruz, CA, USA). 2x Laemmli buffer, Tris buffered saline Tween 20 (TBST) and bovine serum albumin (BSA) were from Sigma-Aldrich, MO, USA. Enhanced chemiluminescence (ECL) kit was from Beyotime, Shanghai, China. TRIzol reagent, PrimeScript™ RT reagent and SYBR Premix Ex Taq kits were from TaKaRa (Dalian, China).

### Experimental design

#### Experiment I

To investigate the effect of ATP on TGF-β-driven activation of Smad-2/3 signaling pathway, the animals (6 rats per group) received a single intraperitoneal dose of ATP, 2 mg/kg [21] for different time intervals (1, 2, 4, 8, 10 and 24 h). Control animals were administered saline (the vehicle). At the indicated time intervals, animals were anesthetized with 50 mg/kg pentobarbital [22] and blood samples were obtained through the retro-orbital plexus. Then serum was separated by centrifugation at 1,000 g for 10 min for measurement of TGF-β. After that, rats were euthanized by means of cervical dislocation. Immediately after death, the kidney was separated, rinsed with PBS (ice-cold) and stored at -80^o^C for determination of p-Smad-2/3 and t-Smad-2/3.

#### Experiment II

Here we examined the role of P2 X 7 receptor in the ATP-induced TGF-β-driven activation of Smad-2/3 signaling. Animals were randomly allocated into 4 groups (6 rats each) and treated intraperitoneally with a single dose of either saline (Control group) or ATP (2 mg/kg) or A 438,079 (3 mg/kg) [23] or A 438,079 one hour before ATP administration. Four hours later (based upon the results of experiment I), animals were anesthetized and blood samples were collected. Then serum was separated for measurement of TGF-β. Rats were then sacrificed and the kidney was separated, rinsed with PBS (ice-cold) and stored at -80^o^C for determination of p-Smad-2/3, t-Smad-2/3.

#### Experiment III

Here we tested whether ATP-induced phosphorylation of Smad-2/3 could be translated into an increase in the expression of pro-fibrotic genes; CTGF and TIMP-1. The implication of P2 X 7 receptor in the ATP-induced pro-fibrotic genes expression was also investigated. The animals were administered either saline or ATP or A 438,079 or A 438,079 in combination with ATP. Twenty four hours later, animals were sacrificed and the kidney was separated, rinsed with PBS (ice-cold) and stored at -80^o^C for the analysis of CTGF and TIMP-1 mRNA and protein expression.

### Assessment of TGF-β_1_ concentration

The serum concentration of TGF-β_1_ was measured using ELISA kit (raised against rat TGF-β_1_) following the manufacturer’s instructions.

### Western blotting

Western blotting analysis was performed to detect p-Smad-2/3, t-Smad-2/3, CTGF, TIMP-1 and β-actin as previously described [24]. Briefly, ice-cold RIBA lysis buffer was used for total protein extraction from kidney homogenates and quantified using Bradford Protein Assay Kit. Protein extracts were denatured by Laemmli sample buffer and separated using 10% sodium dodecyl sulfate (SDS)-polyacrylamide gel electrophoresis and blotted on polyvinylidene fluoride (PVDF) membranes (Millipore, USA). Then, membranes were blocked in TBS-T containing 3% BSA and probed overnight at 4 °C with the appropriate primary antibody followed by an incubation with secondary antibodies (linked to HRP). ECL reagent was used to identify signals in accordance with the manufacturer’s recommendations.

### Real-time PCR

As previously described [24], CTGF and TIMP-1 mRNA levels were determined using real-time PCR.

### Statistical analysis

Data is shown as means ± S.E. Multiple comparisons were made using one-way ANOVA, and Tukey-Kramer was used as a post-hoc analysis. *P*-values less than 0.05 were taken as proof of statistically significant differences between the groups being compared. Statistical analysis was performed using the GraphPad Prism (ISI®, USA) software (version 5).

## Results

### ATP activates serum TGF-β in a time-dependent manner

**Fig. 1:**
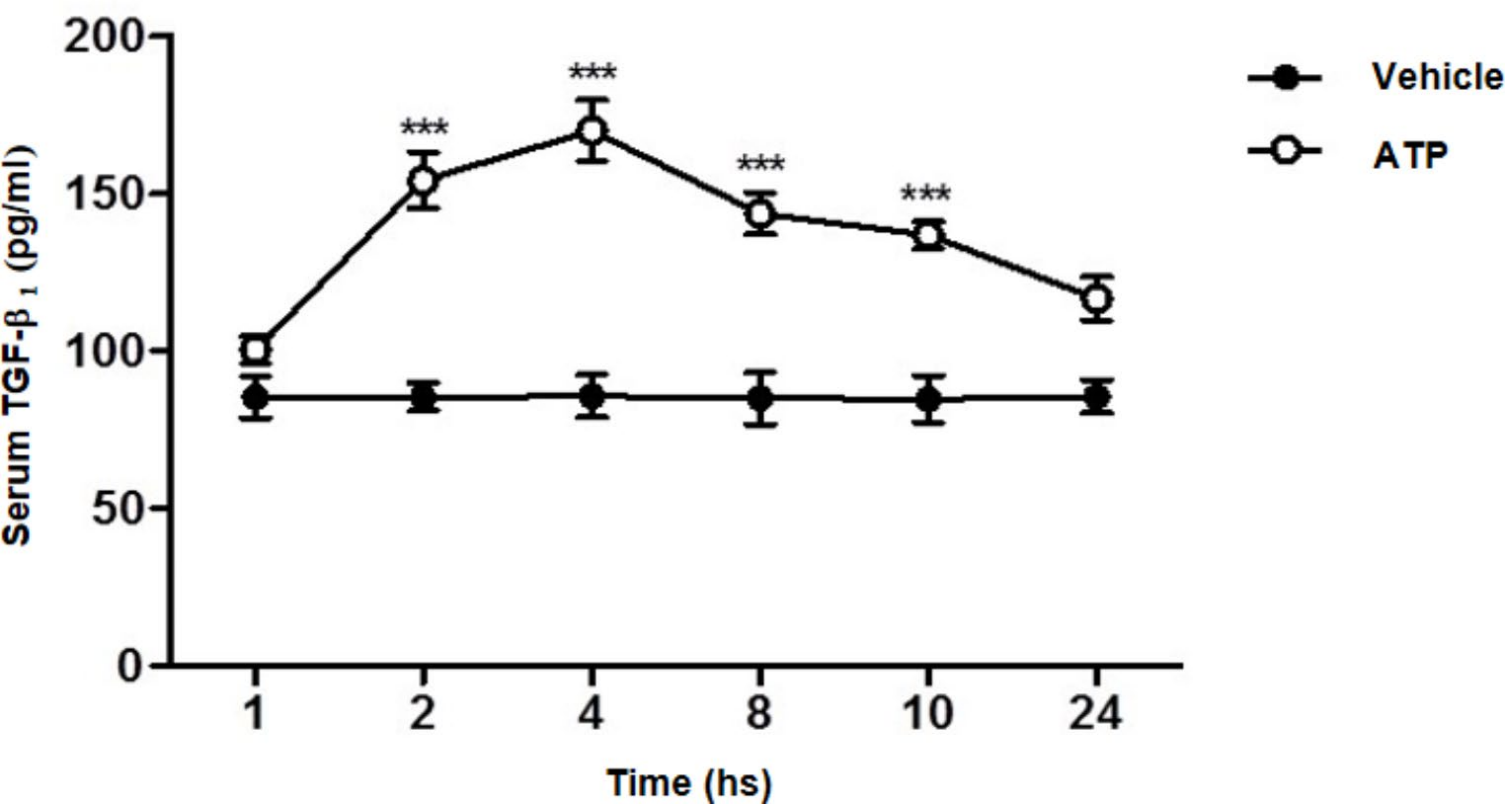
ATP activates TGF-β in a time-dependent manner Serum concentration of TGF-β_1_ was measured by ELISA in rats given either vehicle (Control) or ATP (2 mg/kg i.p.), at 1, 2, 4, 8, 10 and 24 h. Values represent the mean ± S.E. (n = 6), *** *p* < 0.001 when compared to control group

The findings of the current work demonstrated that ATP administration induced a significant activation of serum TGF-β in a time-dependent manner with a peak TGF-β activation recognized reached 4 h after ATP administration (Fig. 1). No significant changes in TGF-β activation were observed in serum of vehicle-treated animals.

### ATP triggers Smad-2/3 phosphorylation in a time-dependent manner

**Fig. 2:**
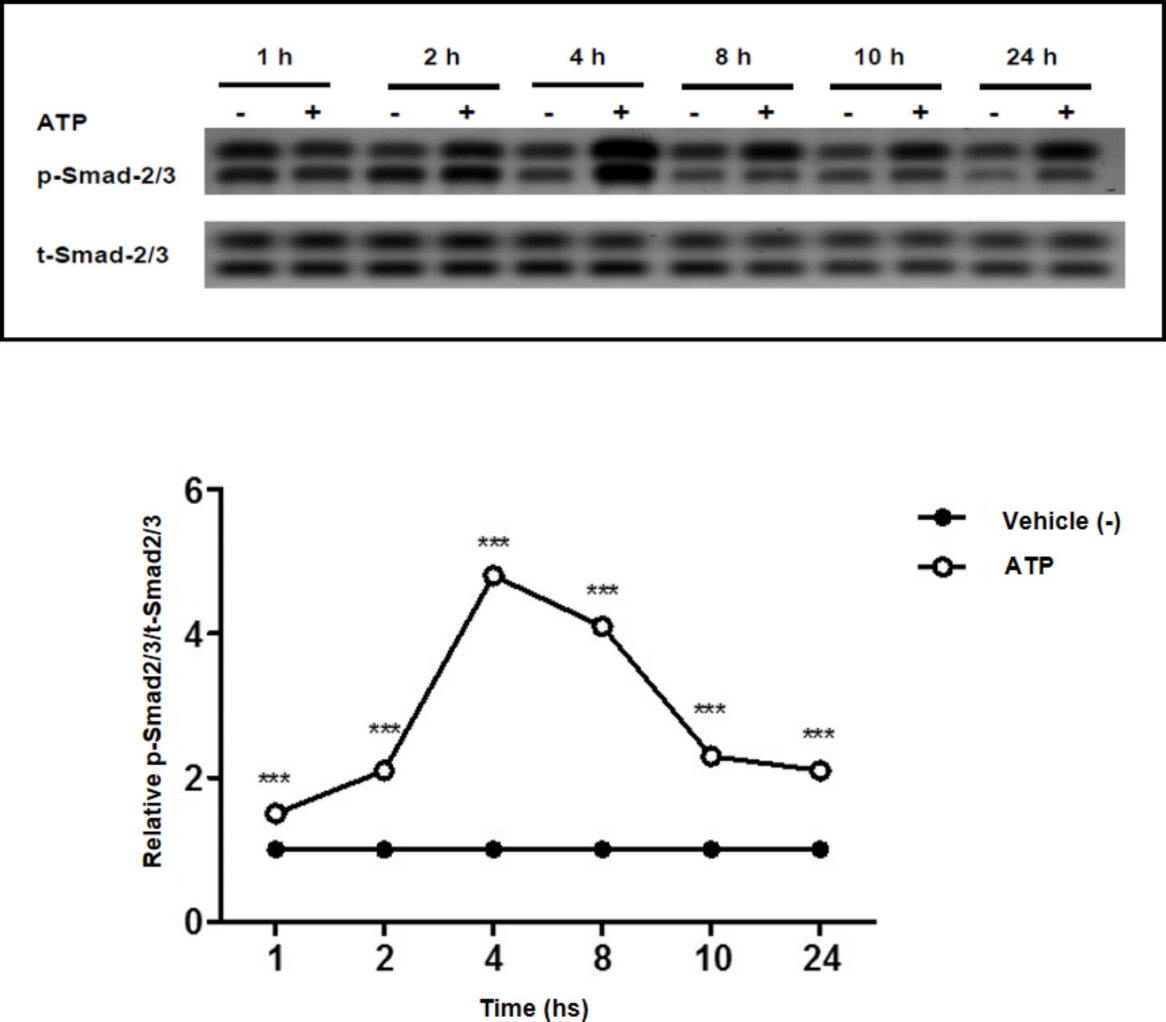
ATP activates Smad2/3 in a time-dependent manner Total kidney extracts were subjected to a Western blot analysis and probed with antibodies against p-Smad-2/3 and total Smads. The rats were given either vehicle (-) or ATP for the indicated time periods. A densitometric analysis of p-Smad-2/3 in relation to the level of total Smads is displayed in the lower panel. Values represent the mean ± S.E. (n = 3), *** *p* < 0.001 when compared to control group

Western blotting analysis of p-Smad-2/3 in kidney tissues from rats treated with ATP showed a significant increase in Smad-2/3 phosphorylation in a time-dependent manner with a peak phosphorylation recognized after 4 h (Peak amount of TGF-β activation) of ATP administration (Fig. 2). These observations indicate that Smad-2/3 activation induced by ATP is critically dependent on TGF-β activation. No significant changes in Smad2/3 phosphorylation were observed in renal tissues of vehicle-treated animals.

### ATP-induced Smad-2/3 phosphorylation is abrogated in the presence of a 438,079

**Fig. 3:**
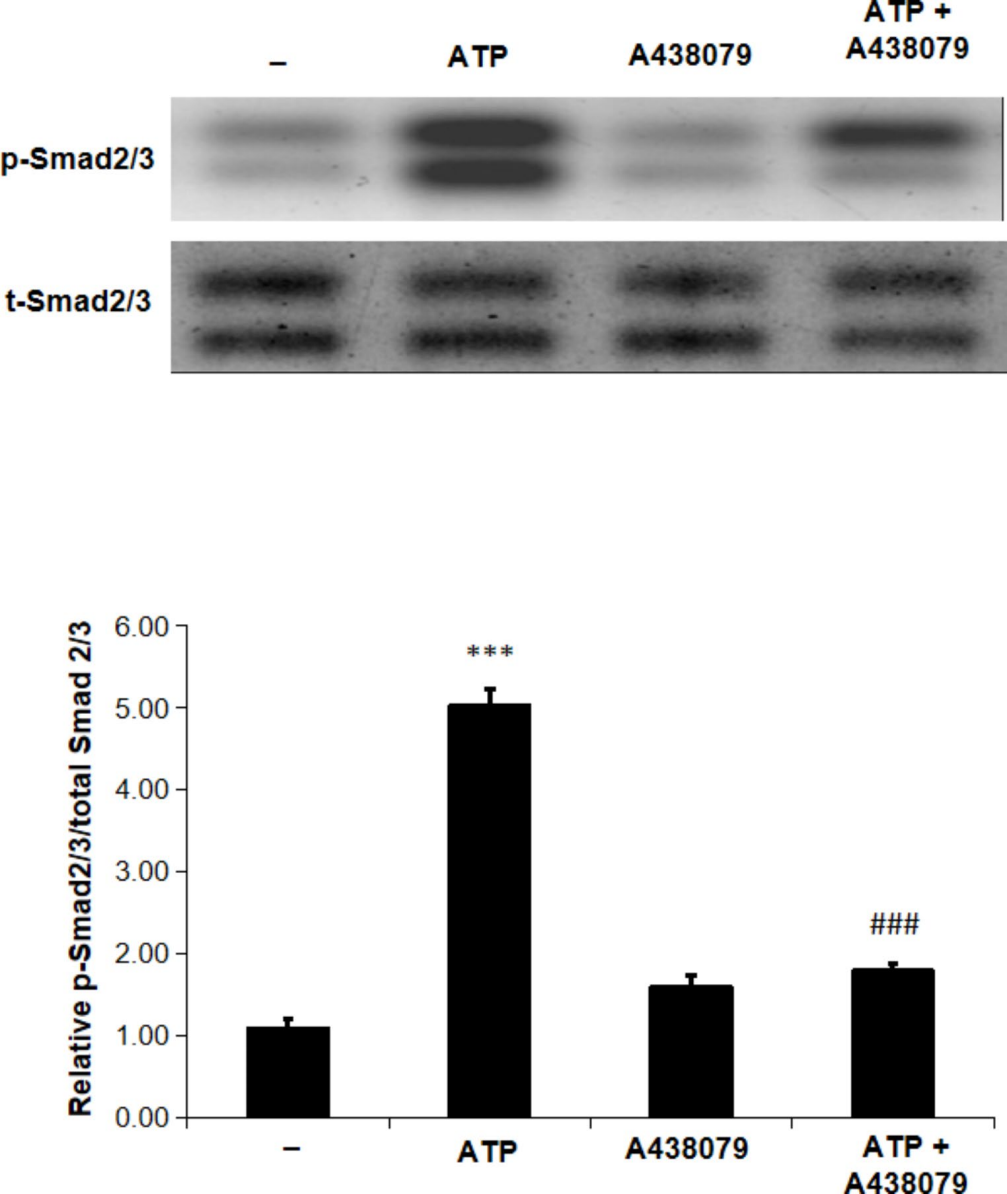
A 438,079 attenuates ATP-induced Smad-2/3 phosphorylation in rat kidney Rat whole kidney extracts were subjected to a Western blot analysis and probed with antibodies against p-Smad-2/3 and total Smads. The animals were given either vehicle (-) or ATP (2 mg/kg i.p.) or A 438,079 (3 mg/kg i.p.) or ATP in combination with A 438,079 for 4 h. A densitometric analysis of p-Smad-2/3 in relation to the level of total Smads is displayed in the lower panel. Values represent the mean ± S.E. (n = 3), *** *p* < 0.001 when compared to control group, and ### *p* < 0.001 versus ATP alone-treated animals

As demonstrated in Fig. 3, administration of ATP significantly increased Smad-2/3 phosphorylation in renal tissues. This increase in Smad phosphorylation is markedly attenuated in animals pre-treated with A 438,079 as compared to ATP alone-treated animals. These findings are specifying the role of P2 X 7 receptor in ATP-triggered Smad-2/3 phosphorylation in rat kidney.

### ATP-induced mRNA transcription of CTGF and TIMP-1 is abrogated in the presence of a 438,079

**Fig. 4:**
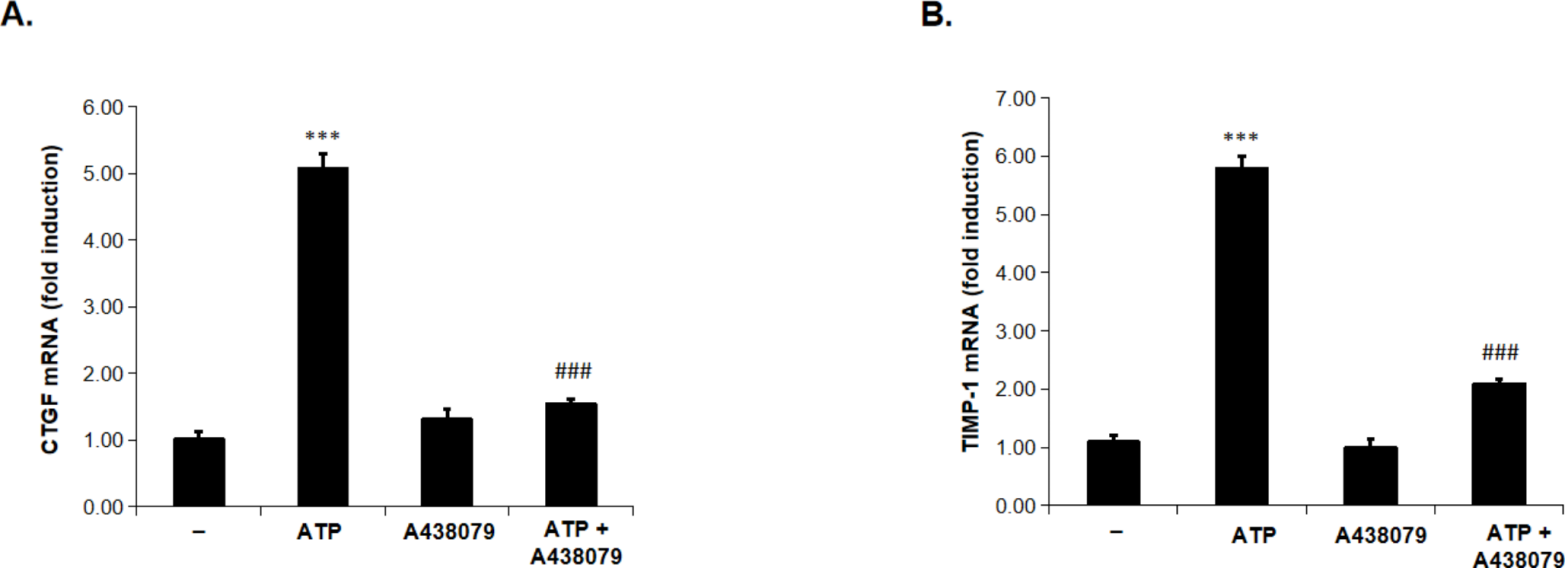
A 438,079 inhibited ATP-induced CTGF and TIMP-1 mRNA transcription in rat kidney Total RNA was isolated from kidney tissues of animals treated with either vehicle (-) or ATP (2 mg/kg i.p.) or A 438,079 (3 mg/kg i.p.) or ATP in combination with A 438,079 for 24 h. The mRNA transcription of CTGF (A) and TIMP-1(B) was determined by Real-time PCR analysis. The mean fold-induction of CTGF and TIMP-1’s mRNA, which was normalized to GAPDH is displayed. Values represent the mean ± S.E. (n = 6), *** *p* < 0.001 when compared to control group, ### *p* < 0.001, against rats treated with ATP alone

As shown in Fig. 4, renal tissues from ATP-treated rats exhibited a significant increase in CTGF and TIMP-1 expression on mRNA level as compared to the control rats. However, mRNA transcription of CTGF and TIMP-1 were significantly reduced in the presence of A 438,079 as compared to ATP alone-treated rats. These findings highlight the role of P2 X 7R in ATP-induced pro-fibrotic changes in renal tissues.

***Protein expression of CTGF and TIMP-1 induced by ATP is abrogated in the presence of A 438,079***.

**Fig. 5:**
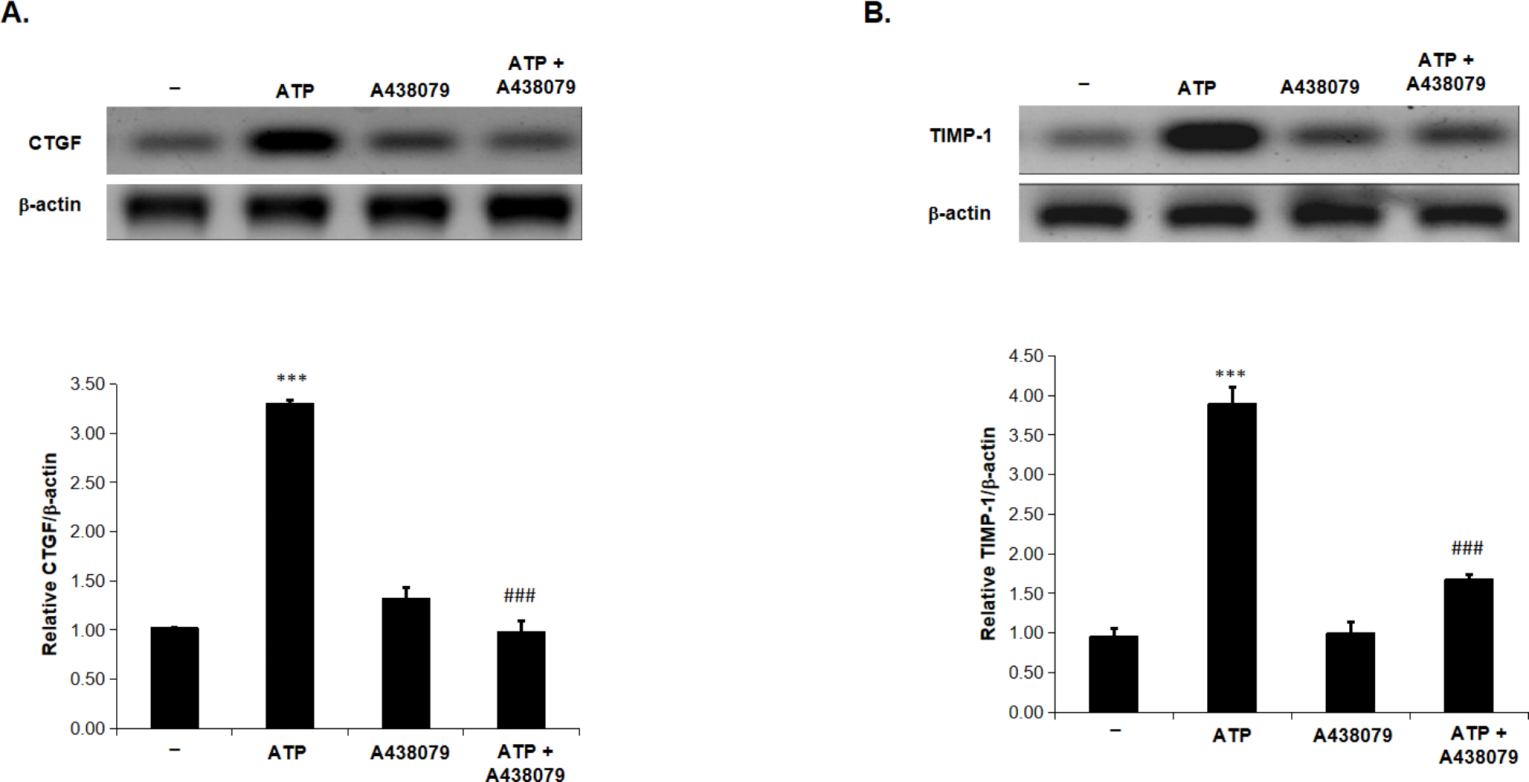
A 438,079 attenuated the protein expression of CTGF and TIMP-1 induced by ATP in rat kidney Total kidney extracts were subjected to a Western blot analysis and probed with antibodies against CTGF (A), TIMP-1 (B), and b-actin antibodies. The rats were given either vehicle (-) or ATP (2 mg/kg i.p.) or A 438,079 (3 mg/kg i.p.) or ATP in combination with A 438,079 for 24 h. A densitometric analysis of CTGF and TIMP-1 in relation to the level of b-actin is displayed in the lower panels. Values represent the mean ± S.E. (n = 3), *** *p* < 0.001 when compared to Control group, and ### *p* < 0.001 versus ATP alone-treated animals

As demonstrated in Fig. 5, CTGF and TIMP-1 protein expression were highly induced in animals treated with ATP compared with control group. On the other hand, this increase in CTGF and TIMP-1 protein expression was significantly reduced in the presence of A 438,079 as compared to ATP alone-treated group, indicating that P2 X 7R is involved in the pro-fibrotic changes induced by ATP in renal tissues.

## Discussion

The basic finding of this study is that ATP administration triggers the expression of the pro-fibrotic genes CTGF and TIMP-1, via activation of the TGF-β-driven Smad-2/3 signaling cascade, an effect that was largely mediated via P2 X 7Rs. A few years ago, the potential role of extracellular ATP as a signal molecule mediating renal inflammation, apoptosis and even fibrosis was confirmed in a mouse model of unilateral ureteric obstruction (UUO), a popular experimental model causing fibrosis [25]. To our knowledge, this is the first study demonstrating the in vivo potential pro-fibrotic effects of ATP in rat kidney. Previously and consistent with our findings, ATP was found to stimulate TGF-β1 mRNA gene expression in rat brain-derived type-2 astrocytes [26]. Binding of ATP to P2 X 7Rs induced Ca influx and K efflux to activate inflammasome with release of IL-1β and, sequentially, IL-1β leading to increased expression of TGF-β [17, 27]. Moreover, strong evidences suggested that activation of P2 X 7Rs was coupled with NADPH oxidase activation and then reactive oxygen species (ROS) and reactive nitrogen species (RNS) generation [28, 29]. Oxidative stress was regarded as one of the major factors involved in TGF-β activation [30].

Transforming growth factor-β has been well-considered as a key mediator of tissue fibrosis [31]. Indeed, it is highly expressed in wide-ranging fibrotic renal injuries [10, 11, 15]. Activation of TGF-β1 is usually accompanied with an increase in Smad-2/3 phosphorylation, the p-Smad-2/3 forms trimeric complex with Smad4 and undergoes nuclear translocation to regulate the transcription of ECM genes. Smad signaling is documented as a major TGF-β downstream signaling pathway activated in progressive tissue fibrosis [32–34], while inhibition of the TGF-β/Smad-2/3 pathway alleviates fibrosis [35]. On the other hand, TGF-β itself could stimulate ATP release into the extracellular spaces, hence amplifying the fibrotic response [36].

TGF-β is usually secreted as latent complex (latent TGF-β) consisting of TGF-β covalently bound to latent TGF-β binding proteins (LTBP) [13, 14]. Activation of TGF-β is achieved by either proteolytic or nonproteolytic events [24]. In the current work, administration of ATP significantly induced TGF-b activation. Importantly, pre-treatment of animals with the P2 × 7Rs antagonist, A 438,079 attenuated the ATP-mediated activation of TGF-β/Smads signaling as demonstrated by the decrease in Smad-2/3 phosphorylation. In parallel, in vivo and in vitro studies showed that inhibition of P2 X 7Rs was linked to the reduction of fibrosis [37, 38]. The ability of TGF-β to activate Smad signaling has a significant role in TGF-β-induced EMT, a process that, when disorganized, plays a major role in a wide array of pathologic disorders, including fibrotic diseases and cancer [39, 40]. Furthermore, we investigated whether ATP-induced Smad-2/3 phosphorylation could be translated into an increase in the expression of the pro-fibrotic genes; CTGF and TIMP-1. It was found that ATP enhanced CTGF expression, an effect that was attenuated by the P2 X 7R antagonist pre-treatment. The pro-fibrotic effect of TGF-β has been mostly mediated via the fibroblast mitogen; CTGF [41, 42]. TGF-β induces CTGF via Smad-binding element whereas the activation of CTGF increases p-Smad-2/3 to promote the transcription of Smad-responsive genes including CTGF itself [43]. In addition, CTGF interacts with epidermal growth factor receptor and induces the phosphorylation of specific tyrosine residues with subsequent activation of intracellular pathways, including the ERK-1/2 [44]. The involvement of ERK activation in the TGF-β-induced CTGF expression has also been documented [45].

In line with our findings, Gazzerro et al. and Panicucci et al. have reported that blockade of P2 X 7 receptor exerts an anti-fibrotic effect and improves muscular function via decreasing concentration of fibrotic mediators as TGF-β and CTGF in experimental models of muscular dystrophies [46, 47]. A growing body of evidence implicates the shift in the balance between matrix metalloproteinases and TIMPs in favor of TIMPs in the excessive deposition of ECM [48]. The present work showed that TIMP-1 mRNA and protein expression was markedly increased in the ATP-treated rats, an effect that was prominently diminished upon treatment with the P2 X 7R antagonist. In harmony with our findings, the pulmonary levels of TIMP-1 were found to increase after intranasal administration of ATPγS in bleomycin treated mice. This increase was extremely reduced in lung tissues in P2 X 7R deficient mice and those treated suramin, a broad-spectrum P2R antagonist [49]. These results shed more light on the role of P2 X 7R in ATP-mediated overexpression of the profibrotic genes CTGF and TIMP-1 that play an important role in ECM deposition and subsequent fibrotic changes in rat kidney.

## Conclusion

**Fig. 6:**
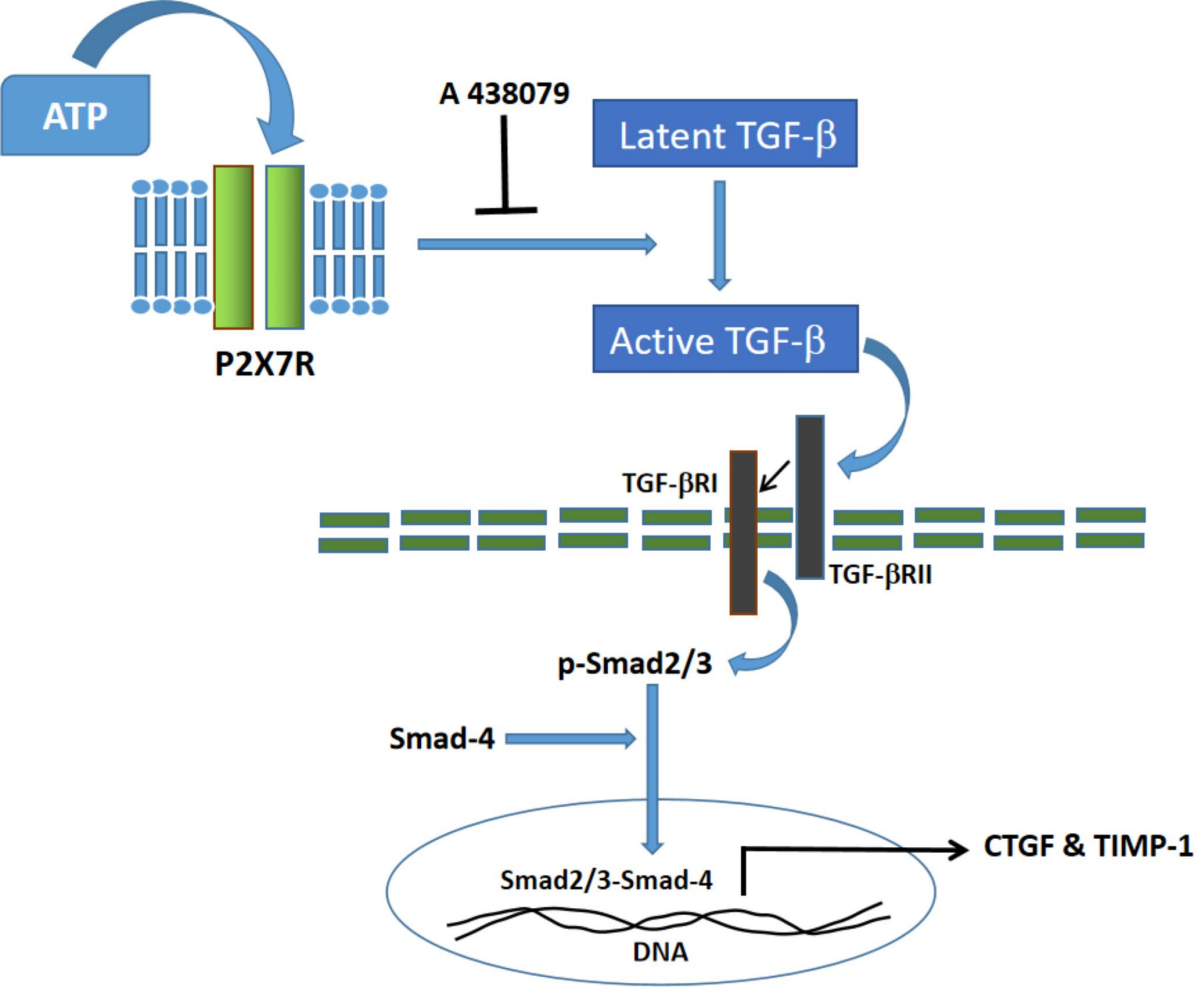
Graphical abstract of the modulatory effect of ATP on the expression of the pro-fibrotic genes CTGF and TIMP-1 in rat kidney Binding of ATP to P2 X 7R triggers a rapid activation of latent TGF-β which in turn activates TGF-β receptors resulting in Smad phosphorylation. Then, phosphorylated Smads (p-Smad2/3) form a complex with smad-4 which moves into the nucleus and stimulate the expression of the profibrotic genes CTGF and TIMP-1. Adenosine 5′-triphosphate (ATP); The purinergic P2 X 7 receptor (P2 X 7R); A specific antagonist of P2 X 7 receptor (A 438,079); Connective tissue growth factor (CTGF); Tissue inhibitors of matrix metalloproteinases-1 (TIMP-1); Transforming growth factor-β (TGF-β); Transforming growth factor-β receptor I (TGF-βRI); Transforming growth factor-β receptor II (TGF-βRII).

Overall, our data shows that ATP regulates pro-fibrotic events in the kidney via the TGF-β-driven Smad-2/3 signaling cascade and subsequent expression of the pro-fibrotic genes CTGF and TIMP-1, an effect that was largely mediated via P2 X 7R (Fig. 6). Clinical studies are needed to investigate the potential role of ATP in renal fibrosis.

### Electronic supplementary material

Below is the link to the electronic supplementary material.

Supplementary Material 1

Supplementary Material 2

Supplementary Material 3

Supplementary Material 4

Supplementary Material 5

Supplementary Material 6

## Data Availability

Data will be made available on request.
