# Supplementary figures and images for: P2 X 7 receptor is a critical regulator of extracellular ATP-induced profibrotic genes expression in rat kidney: implication of transforming growth factor-β/Smad signaling pathway
Source: Purinergic Signal. 2023 Nov 7;20(4):421–30. doi: 10.1007/s11302-023-09977-4 (PMC11303607; doi:10.1007/s11302-023-09977-4)

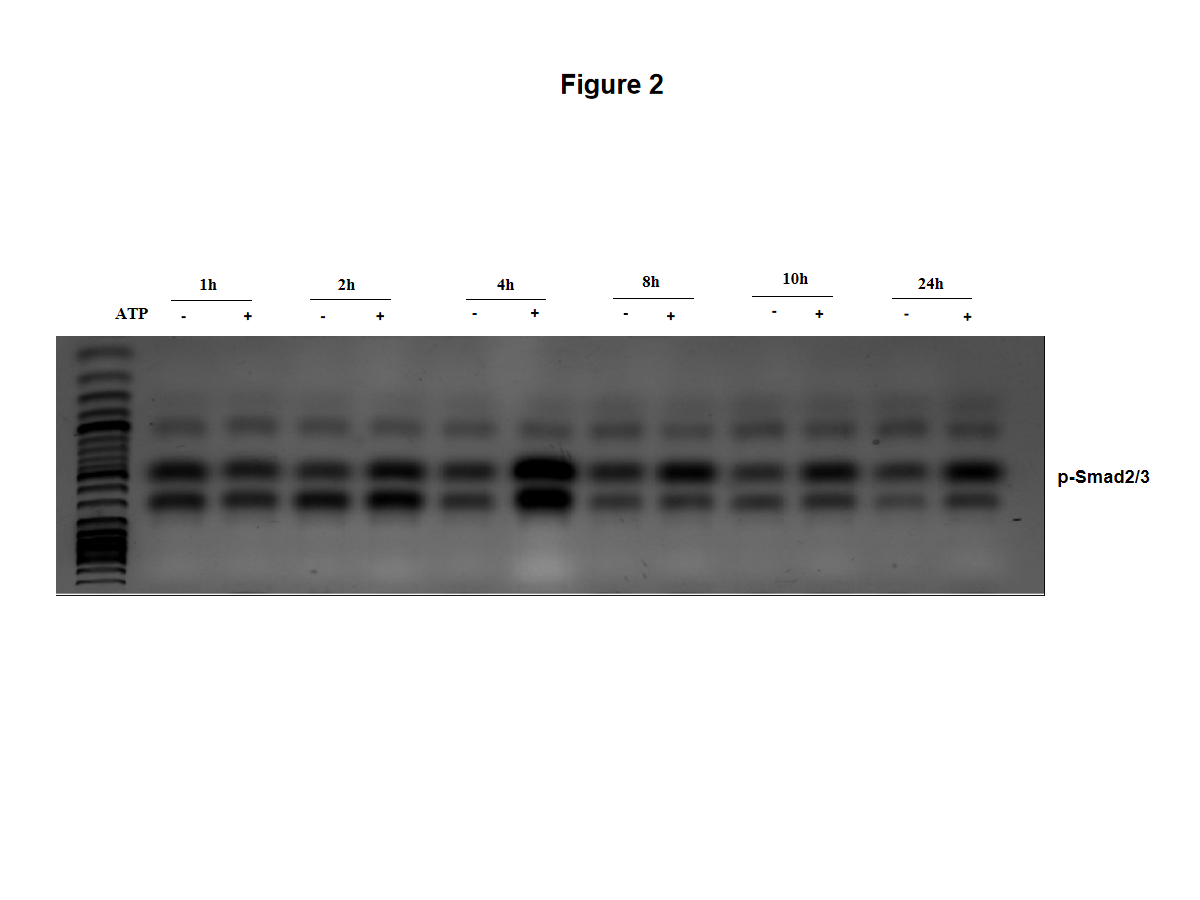

Supplement: Supplementary file 1 — Supplementary Material 1 [file 11302_2023_9977_MOESM1_ESM.tif]

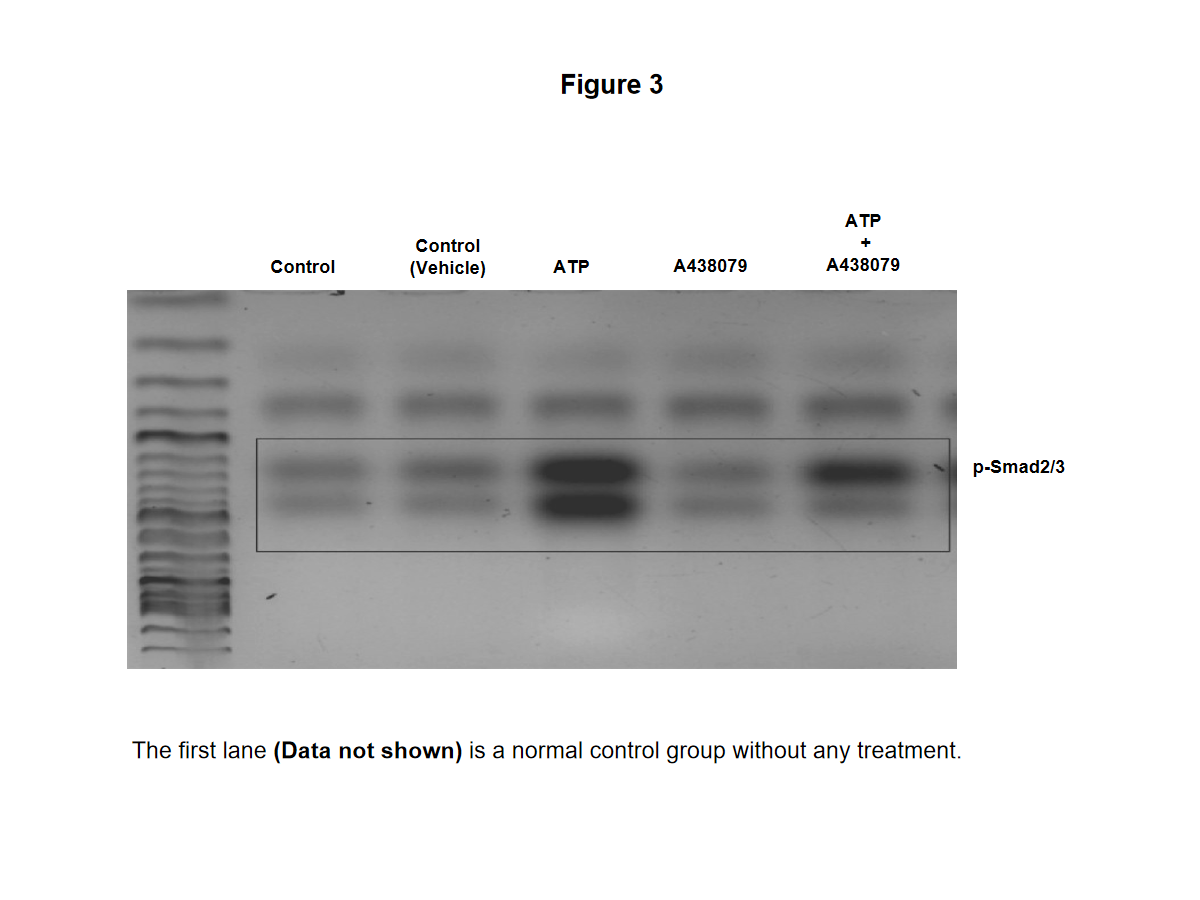

Supplement: Supplementary file 2 — Supplementary Material 2 [file 11302_2023_9977_MOESM2_ESM.tif]

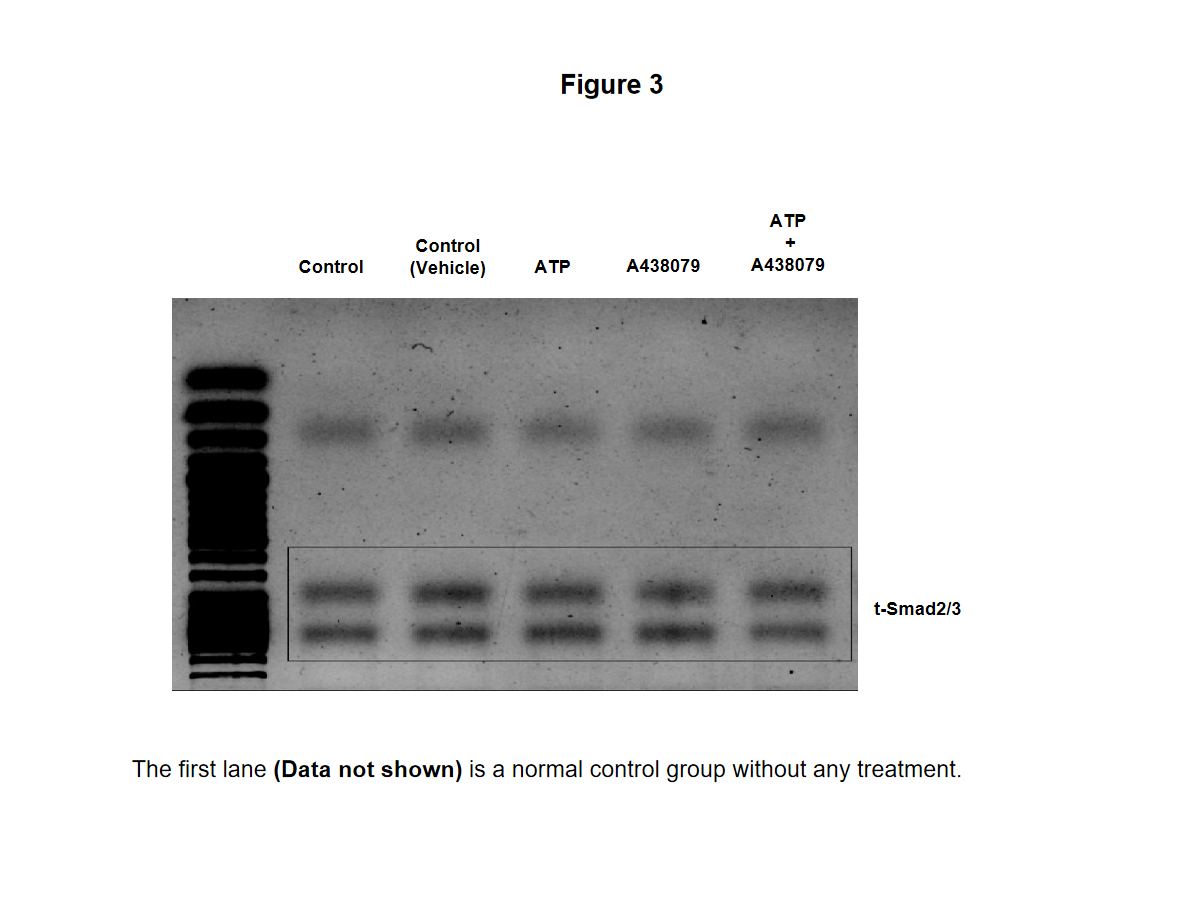

Supplement: Supplementary file 3 — Supplementary Material 3 [file 11302_2023_9977_MOESM3_ESM.tif]

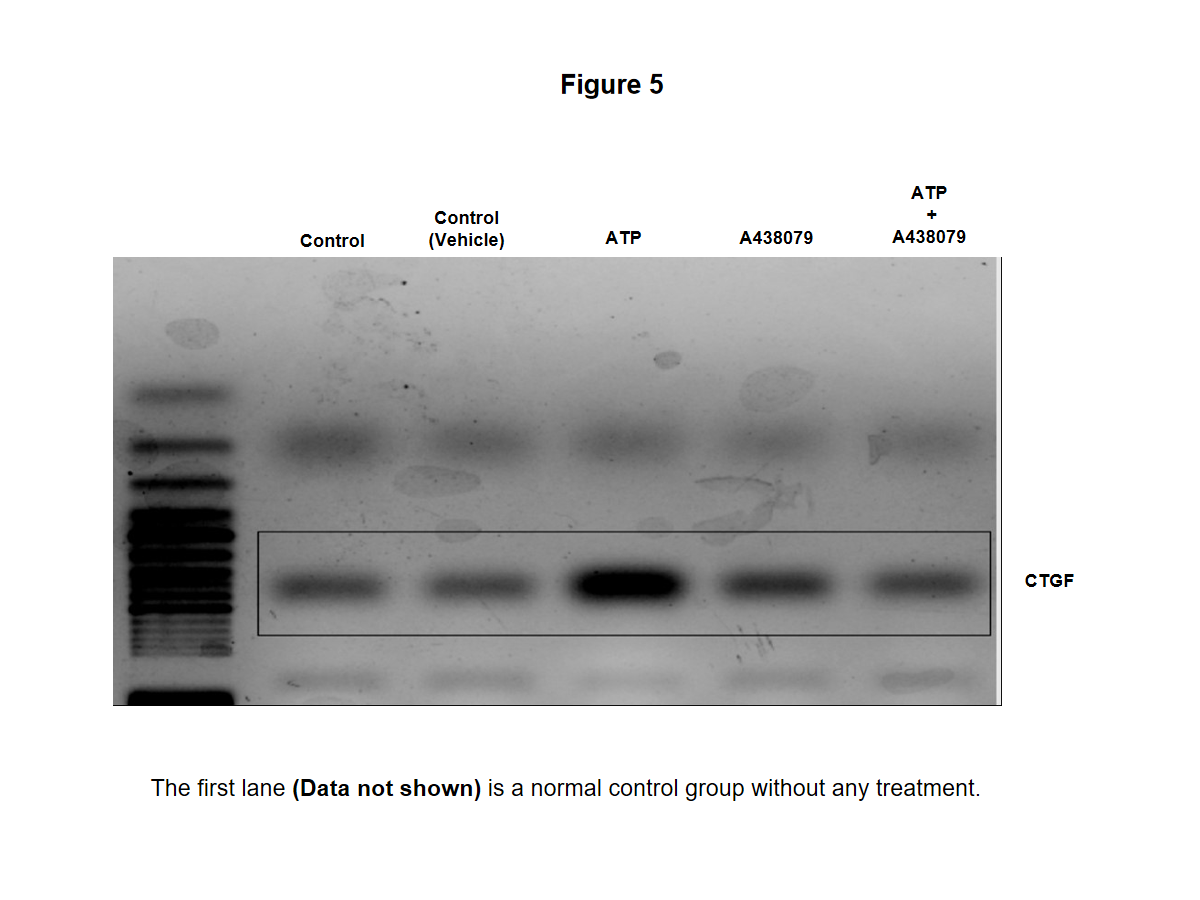

Supplement: Supplementary file 4 — Supplementary Material 4 [file 11302_2023_9977_MOESM4_ESM.tif]

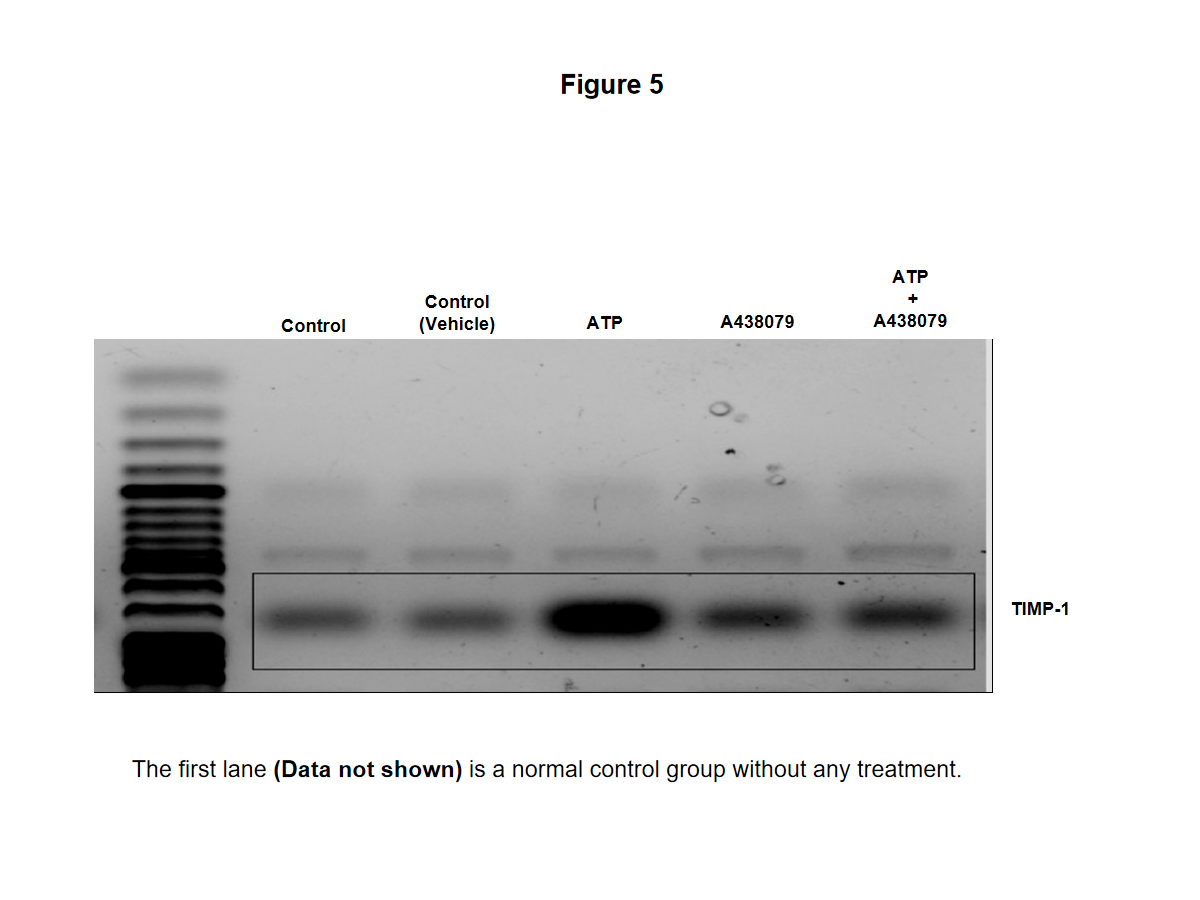

Supplement: Supplementary file 5 — Supplementary Material 5 [file 11302_2023_9977_MOESM5_ESM.tif]

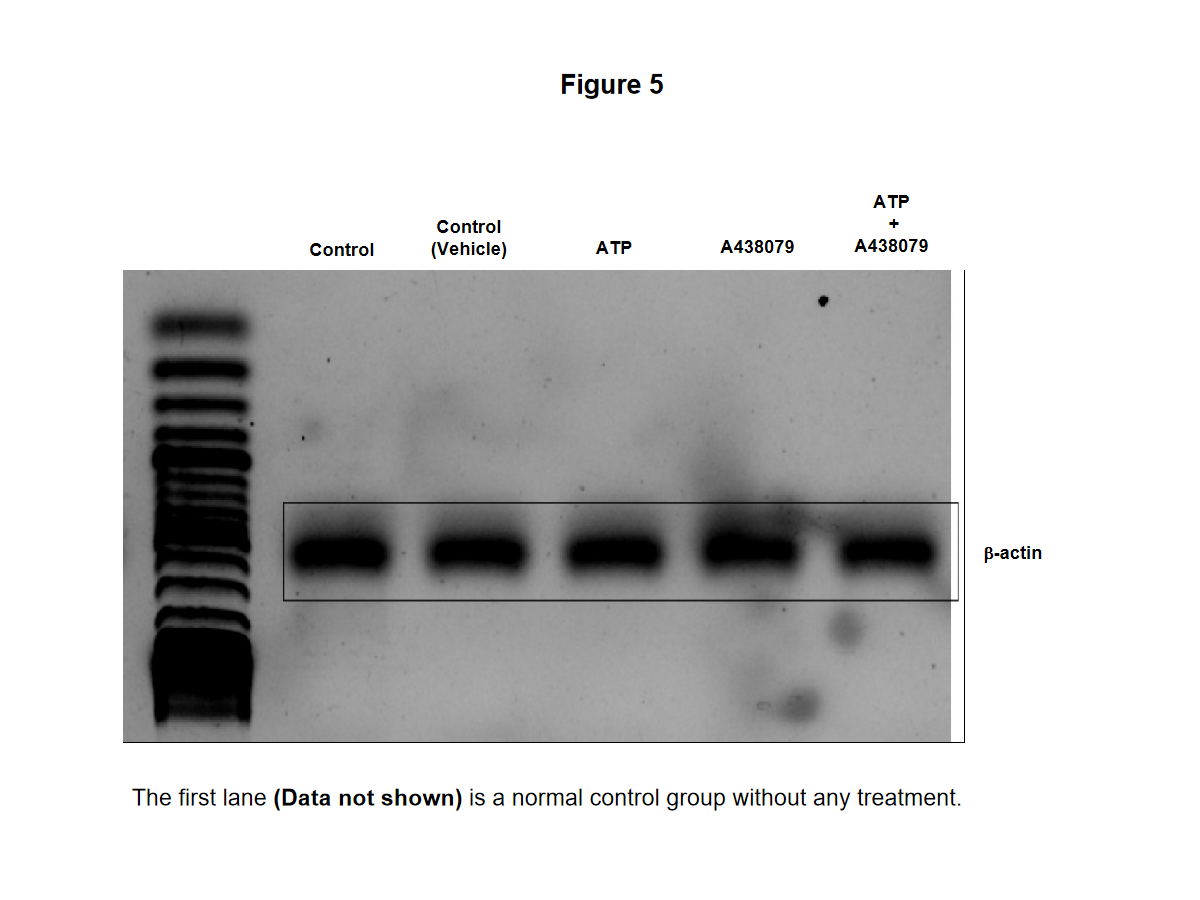

Supplement: Supplementary file 6 — Supplementary Material 6 [file 11302_2023_9977_MOESM6_ESM.tif]
